# Global Convergence on the Bioethics of Surgical Implants

**DOI:** 10.1155/2015/853125

**Published:** 2015-04-20

**Authors:** Alberto Garcia, Dominique J. Monlezun

**Affiliations:** ^1^UNESCO Chair in Bioethics & Human Rights, Rome, Italy; ^2^School of Bioethics, Athenaeum Pontificium Regina Apostolorum, Rome, Italy; ^3^Tulane University School of Medicine, New Orleans, LA, USA

## Abstract

The increasing globalization of mankind with pluralistic belief systems necessitates physicians by virtue of their profession to partner with bioethics for soundly applying emerging knowledge and technologies *for the best use of the patient*. A subfield within medicine in which this need is acutely felt is that of surgical implants. Within this subfield such recent promising ethics and medicine partnerships include the International Tissue Engineering Research Association and UNESCO Chair in Bioethics and Human Rights' International Code of Ethics. In this paper, we provide an overview of the emerging human rights framework from bioethics and international law, discussion of key framework principles, their application to the current surgical challenge of implantation of surgical mesh for prolapse, and conclusions and recommendations. Such discussions are meant to facilitate true quality improvement in patient care by ensuring the exciting technologies and medical practices emerging new daily are accompanied by an equal commitment of physicians to ethically provide their services for the chief end of the patient's good.

## 1. Introduction

“The art [of medicine] consists of three things: the disease, the patient, and the physician. The physician is the servant of the art, and the patient must combat the disease along with the physician.”—Hippocrates, De morbis popularibus 1.2.

Since the codification of medicine as a science and an art by the Greek Hippocrates in 4th century BC, the increasing globalization of mankind with pluralistic belief systems has enriched, and complicated, this discipline. True to modern medicine's earliest principles articulated by the Hippocrates Oath [1]—justice, patient confidentiality, respect for teachers, and solidarity with peers—this diverse international environment necessitates physicians by virtue of their profession to partner with bioethics for soundly applying emerging knowledge and technologies* for the best use of the patient. *A subfield within medicine in which this need is acutely needed is that of surgical implants. Few fields are as rapidly growing in technical complexity and ethical challenges as surgery, in which advances in genomic and bioengineering (including nanotechnology) daily push the boundaries of human possibility. Professor Mueller notes how professional ethics runs through this field as noted with The European Board of Urology (EBU) European Curriculum for Urology [2]. He highlights how this curriculum highlights the technical limitation of medicine as it can teach the information for emergency and elective cases, but the “need to act at all times in an ethical and professional manner” is required to understand how to pragmatically apply this information. Such dual competencies of physicians in the technical aspect of medicine with the art of its application amplify the “importance and understanding of evidence-based medicine,” particularly with the correlate that limited evidence for a new innovation of various surgical implants should bid practitioners caution. In this paper, we provide (Section 2) an overview of the emerging human rights framework from bioethics and international law, (Section 3) discussion of key framework principles, (Section 4) their application to the current surgical challenge of implantation of surgical mesh for prolapse, and (Section 5) conclusions and recommendations.

## 2. Human Rights Framework from Bioethics and International Law

A promising bioethics resource for surgeons is the growing global consensus of human rights not only as commonly beliefs supported by diverse belief structures, but also as core bioethics tenants and international law, since the Universal Declaration of Human Rights adoption in 1948 by the United Nations (UN) in the World War II aftermath [3]. The United Nations Educational, Scientific, and Cultural Organization (UNESCO) Declaration on Bioethics and Human Rights (DBHR) in 2005 built on this worldwide consensus by its historic commitment through its 193 UN member states to apply 15 defined bioethical principles within and across countries [4]. Following such international human rights violations in the 1990s as the US-led placebo-control HIV study in Africa [5], the UNESCO Declaration emerged as an attempt to synthesize human rights and bioethics through its approach, both flexible enough to allow specific situation application and yet broad enough to allow pluralistic consensus, particularly in almost half of UN states without national bioethics committees to advise their governments [6].

We thus propose the following directly relevant principles from this approach as an ethically robust and pragmatically useful framework for exploring and converging on protocols for the optimal patient application of surgical implants: (I) human dignity and human rights, (II) benefit and harm, (III) autonomy and consent, and (IV) justice. Unless such an interdisciplinary approach with a global scope to medicine is adopted, then the shared expertise of physicians (including those from multiple disciplines), scientists, lawyers, philosophers, and patients from varied belief backgrounds cannot reach concrete and substantive protocols. And without such protocols, as the operationalized products of the internationally supported bioethical principles, medicine cannot realize its end as a science and art at service of the patient. “Fostering the art of convergence and cooperation in global ethics” as the mission statement of the UNESCO Chair in Bioethics and Human Rights [7] can thus become the necessary complementary art to medicine in the modern world.

## 3. Bioethics Principles in a Human Rights Framework

(I)* Human dignity and human rights *as the first DBHR principle provides a foundation for the remainder by asserting the primacy of the individual's interests and welfare over the interests of the society or scientific community. This prioritization is based up on the human dignity of each individual and his or her derivative rights and freedoms flowing from it. Though the DBHR excludes any defined justification of this first principle and its normative nature dictating right and wrong actions toward any individual, this omission allows for the substantive convergence of different peoples to acknowledge and anchor this fundamental principle from within their own belief systems.

(II)* Benefit and harm* describes how the operationalization of the first principle entails seeking the preferred balance between benefit and harm for the individual. Similar to the original Hippocratic Oath with its insistence to “do no harm,” DBHR asserts that the minimization of harm and the maximization of direct and indirect benefits for individual much be sought during the advancement of scientific knowledge and its medical practice and technologies.

(III)* Autonomy and consent* are the related principles that define the parameters necessary for physicians' right relationships with patients. Physicians according to the autonomy principle thus respect a patient with adequate capacities and conditions (i.e., maturity and freedom from external or internal coercion) to make decisions and respond with responsibility to their effects. Consent is the derivative duty for physicians and researchers in light of patient's autonomy to provide them the information and absence of coercion to make a free and prior decision for any medical intervention or research project to be provided to them (or withdrawn from them at a later time).

(IV)* Justice* describes the social dimension of the fundamental principle of human rights by emphasizing the equality among all individuals by virtue of the equivalence of their dignity and rights. The correlate duty for physicians and researchers is to thus treat them equally. This principle of justice is further elaborated as distributive justice by the associated principles of nondiscrimination, respect for cultural diversity and pluralism, solidarity, and sharing of benefits of scientific research.

## 4. Application for Surgical Mesh Bioethics Analysis

### 4.1. Clinical Background

Pelvic organ prolapse (POP) occurs in women when such pelvic organs as the bowel, rectum, uterus, or bladder fall against the vaginal wall from their usual anatomical positions, when the internal walls that hold these organs are compromised. Quality of life can decrease with this condition, along with altered sexual and urinary function. Urogynecologic surgical mesh is a medical device for transvaginal repair of POP by strengthening these damaged or weakened internal structures [8]. In April of 2014, the United States Food and Drug Administration (FDA) proposed two orders to require mesh manufacturers to submit to the FDA a premarket approval application providing clinical data supporting the device's effectiveness and safety, in addition to reclassifying mesh from a class II device (moderate-risk) to class III (high-risk) [9].

These orders followed a multiyear FDA investigation and independent removal of such particular mesh products as Protogen-Sling from the market after increasing complication rates, including vaginal mesh erosions in 10% of cases that can then lead to infections, nerve lesion-based chronic pain, sepsis, perforated organs, and death. This American government regulatory agency approved this product without clinical testing [10] and thus allowed multiple POP meshes to be approved and applied for human patients based on the original 1997 Protogen-Sling approval. From 2008 to 2010, there were 1503 POP complication reports submitted to the FDA, and the first patient settlement made in July of 2012 totaled $5.5 million in the multidistrict litigation (MDL)-2187 against the device company, Bard [11].

### 4.2. Dangers of Lawsuit and Market-Based Patient Care Improvement

The necessity for the institutionalization of bioethics and medicine's partnership, and thus the timely application of bioethics analysis of the above principles for optimal patient care, is demonstrated in the legal and regulatory failure for POP patients. This can be seen with the time delays and resulting unnecessary patient suffering (and economic loss from ethically suspect device companies) with the first market withdrawal of the Protogen-Sling in 1999, bending to market pressures from the escalating complication rates. Yet a decade lapsed before the FDA in September of 2011 mandated 34 POP mesh manufacturers to conduct clinical retrospective studies for product evaluation, one year before the first patient lawsuit settlements began. The urgent question arising from this unfortunate situation is not only were the manufacturing companies or physicians unethically promoting untested and unsafe products for patients, but why did it require over 10 years of legal and market pressures to improve patient care? Thus the following bioethics analysis is provided not simply as an academic exercise for select experts, but rather as a needed* first and ongoing step* in the medical science development and clinical settings.

### 4.3. Bioethics Analysis as True Quality Improvement

To have ensured the true quality improvement in POP care, the following bioethics analysis could have (and should for future products) be applied in light of the human rights-framework of bioethics principles: (I) human dignity and human rights, (II) benefit and harm, (III) autonomy and consent, and (IV) justice. Beginning with the first principle, the (I) human dignity and thus rights of POP patients would have implicated the FDA and early physician adopters of POP mesh in the required systematic and detailed scientific and clinical analysis of the new products to ensure their safety and clinical effectiveness. The medical community and its regulatory bodies including the FDA owe their patients a commitment to prioritizing their health over industries who can capitalize at worst, or fail to adequately consider at best, patients' distress stemming from their disease states that can drive them to seemingly quick medical solutions. Dr. Pellegrino, the father of modern bioethics, discusses the patients' exploitable state by asserting that the three aspects of medicine are a natural conclusion from the fundamental assumption of each patient's dignity [12]. Namely, (i) the disease makes the patient vulnerable, (ii) the physician by his or her nonproprietary medical knowledge must use such knowledge to respond competently and compassionately to the patient's vulnerable state, and (iii) the oath-based professional and public commitment of the physician binds them to honor the patient-physician relationship with faithfulness to its goal of the patient's health above all other interests.

From this ethical starting point, the next bioethics principle of (II) benefit and harm highlights the medical community and regulatory agencies' duty—as the logical consequence of patients' dignity—to make unbiased judgments from the scientific and clinical analysis of the POP devices. These judgments must seriously question if implanting a semipermanent or permanent device into a patient should be done with regulatory agency approval without such implantation ever being done before in a human. The judgment of the FDA and early physician adopters of the Protogen device, for instance, appeared to be that the benefits of putting this device on the market for patients outweighed the potential harms that were not adequately analyzed or understood before their use was begun [9]. Due to the complex and ever-evolving nature of medical treatment and devices' developments, the limited knowledge of POP patients (and patients in general) to make their own judgment on benefits and harms is thus compromised if physicians and scientists fail in this duty.

Physicians in response to the next bioethics principle of (III) autonomy and consent therefore are bound by the patient-physician relationship with its roots in the patients' fundamental dignity to properly assess, understand, and communicate in an unbiased and nondirective way the benefits and harms for a patient's consent to validly be given. Patients with adequate capacities and necessary conditions (i.e., reasonably free of pressure from significant others) thus must have their autonomy respected by physicians to have open, free, and understandable discussions about POP meshes to determine if their consent should be given or delayed until more proven devices are demonstrated. Because POP is not a life-threatening condition, it is questionable if physicians can claim inadequate time exists for them to critically review the state of knowledge regarding the emerging devices, consult with regulatory agencies and professional medical societies, and make recommendations for their patients to allow them truly informed consent.

Finally, application of the bioethics principle of (IV) justice emphasizes the importance of ethical device development and its application across social strata. For a mesh to require over 9 operative revisions as noted in the American lawsuits [10], the medical care costs for such repeated mesh surgeries can be prohibitive and thus only available to higher socioeconomic populations. Smarter drug and device design on the part of the manufacturers may ensure a more equitable distribution of these treatments. On the part of the physicians applying the devices, adherence to this justice principle may provide the ethical argument needed to motivate the medical community to cost efficiently assist patients in the preventive management of their diseases or disease risks such that more costly surgeries such as POP mesh implementation may be more justly applied for the conditions necessitating them. Societal resources thus may be freed up for more just distribution more on a need-basis rather than a wealth-basis. Physicians cannot expect legal or market pressures to do their jobs for them—namely, advocating for the health of the patient by caring justly and competently for them, rather than leaving them to seek recourse through the courts or shopping around for different medical treatments.

## 5. Conclusions and Recommendations

Our world's globalization and rapid medical technology development must catalyze a global ethics and medicine. Amid diverse belief systems, convergence as well as cooperation is not only possible across patients, physicians, regulatory agencies, and industries as seen in the growing body of international law and human rights declarations with multinational support—they are necessary. The case of surgical mesh for pelvic organ prolapse typifies the failure of regulatory agencies and physicians to honor their identity as servants of the science and art of medicine, at service of their patients, if they fail to discharge their duty for the art of ethical convergence throughout device and drug development and their deployment to patients.

We have proposed the international consensus-based human rights approach to bioethics to facilitate optimal medical technology and practice evolution through the partnership between science and ethics. Since being the world's medical expert in surgical mesh does not make one an ethical expert in their development or use, collaboration is a necessary dimension of medicine to ensure that patients do not bear the harmful consequences of poorly tested technologies. Such ethical partnership from the beginning of technology development and ongoing through its practice in medicine is thus an integral aspect of quality improvement to ensure that evidence-based medicine includes the ethical base as well. The fear of stifling medical innovation or market growth is thus invalid as there are no shortcuts to patient care, including skipping bioethics steps that must accompany the development of the science and art of medicine.

Such recent ethics and medicine partnerships as the International Tissue Engineering Research Association and UNESCO Chair in Bioethics and Human Rights' International Code of Ethics [13] suggest a promising route forward. Such partnerships may help ensure the patient's case be resolved in their exam room, not in a legal court or stock market. Therefore, the exciting technologies and medical practices emerging new daily must be accompanied by an equal commitment of physicians to ethically provide their services for the chief concern of the patient's good. Hearkening to Hippocrates, physicians must combat the disease with their patients through ongoing and rigorous medical and bioethical reflection—lest the physician or patient, rather than the disease, become the enemy the other must combat.
